# Swim Speed, Behavior, and Movement of North Atlantic Right Whales (*Eubalaena glacialis*) in Coastal Waters of Northeastern Florida, USA

**DOI:** 10.1371/journal.pone.0054340

**Published:** 2013-01-10

**Authors:** James H. W. Hain, Joy D. Hampp, Sheila A. McKenney, Julie A. Albert, Robert D. Kenney

**Affiliations:** 1 Associated Scientists at Woods Hole, Woods Hole, Massachusetts, United States of America; 2 Marineland Right Whale Project, Marineland, Florida, United States of America; 3 Marine Resources Council, Palm Bay, Florida, United States of America; 4 Graduate School of Oceanography, University of Rhode Island, Narragansett, Rhode Island, United States of America; Institut Pluridisciplinaire Hubert Curien, France

## Abstract

In a portion of the coastal waters of northeastern Florida, North Atlantic right whales (*Eubalaena glacialis*) occur close to shore from December through March. These waters are included within the designated critical habitat for right whales. Data on swim speed, behavior, and direction of movement – with photo-identification of individual whales – were gathered by a volunteer sighting network working alongside experienced scientists and supplemented by aerial observations. In seven years (2001–2007), 109 tracking periods or “follows” were conducted on right whales during 600 hours of observation from shore-based observers. The whales were categorized as mother-calf pairs, singles and non-mother-calf pairs, and groups of 3 or more individuals. Sample size and amount of information obtained was largest for mother-calf pairs. Swim speeds varied within and across observation periods, individuals, and categories. One category, singles and non mother-calf pairs, was significantly different from the other two – and had the largest variability and the fastest swim speeds. Median swim speed for all categories was 1.3 km/h (0.7 kn), with examples that suggest swim speeds differ between within-habitat movement and migration-mode travel. Within-habitat right whales often travel back-and-forth in a north-south, along-coast, direction, which may cause an individual to pass by a given point on several occasions, potentially increasing anthropogenic risk exposure (*e.g*., vessel collision, fishing gear entanglement, harassment). At times, mothers and calves engaged in lengthy stationary periods (up to 7.5 h) that included rest, nursing, and play. These mother-calf interactions have implications for communication, learning, and survival. Overall, these behaviors are relevant to population status, distribution, calving success, correlation to environmental parameters, survey efficacy, and human-impacts mitigation. These observations contribute important parameters to conservation biology, predictive modeling, and management. However, while we often search for predictions, patterns, and means, the message here is also about variability and the behavioral characteristics of individual whales.

## Introduction

As early as the 1950s, researchers reported the seasonal occurrence of North Atlantic right whales, *Eubalaena glacialis*, in Atlantic coastal waters of the southeastern United States (SEUS—here defined as south of the South Carolina/Georgia border), with the suggestion that the area was a calving ground for the population [1]–[4]. Accumulating evidence led to an early effort in 1982–83 to develop a right whale sighting network in the SEUS [5]. In 1984, the first aerial surveys for right whales in the SEUS (by a volunteer group of commercial airline pilots) commenced [6], [7]. Sighting data collected between 1950 and 1989 were synthesized to define three proposed critical habitats, including the SEUS, for right whales under the Endangered Species Act [8], which were subsequently designated by the National Marine Fisheries Service on 3 June 1994 [9].

The SEUS right whale critical habitat extends from 31°15′ N latitude (off St. Simon's Island, Georgia) to 30°15′ N (between Jacksonville Beach and Ponte Vedra Beach, Florida) and out to 15 nautical miles (nm) (27.8 km) offshore; and, in a narrower section, from 30°15′ N to 28°00′ N (off Melbourne Beach, Florida) out to 5 nm (9.3 km). The SEUS critical habitat is a total of 215 nm (398 km) in latitudinal extent, with 175 nm (324 km) in Florida, and 140 nm (259 km) south of 30°15′ (the point at which the defined habitat narrows). South of 30°15′, the inshore isobaths approach the shoreline and the nearshore zone of shallow waters (<33 ft or 10 m) narrows. In the SEUS, right whale distribution is concentrated in water depths between 10 and 20 m [10]. To the north of 30°15′ these depths (and most right whales) are several km from shore (nearshore waters are shallower). South of 30°15′, the whales' preferred depths (and some right whales) are often within ½ nm (1 km) from shore. The nearshore seafloor has a generally shallow slope, and features a breaker zone, trough, and an offshore bar – all of which are changeable seasonally and between periods of winter and summer storm and calm [11]. While sea conditions are generally warm and calm [12], a tongue of colder water occurs adjacent to shore in winter [13], [14]. This too is variable in temporal and spatial occurrence.

Protection of the species and the habitat has continued to evolve. The majority of anthropogenic right whale deaths are due to collisions with ships [15]. Therefore, management priority has focused on the portion of the SEUS critical habitat with the combination of greatest right whale density and most frequent vessel traffic, as well as the location of three major ports with associated channel entrances:

* Brunswick River and the Port of Brunswick, Georgia;
* St. Marys River and the ports of Kings Bay, Georgia, and Fernandina Beach, Florida;
* St. Johns River and the ports of Mayport and Jacksonville, Florida.

An Early Warning System (EWS) to advise mariners of whale locations was established in 1994, and a Mandatory Ship Reporting (MSR) System was designated in 1999 [7], [16]–[18]. (The more southerly Port Canaveral, at latitude 28°25′, while within the critical habitat, is not included in the EWS or MSR.) In addition to activities directly related to reduction of human impacts on right whales, recovery-plan implementation objectives include characterization, monitoring, and protection of important habitat; and identification and monitoring of the status, trends, distribution, and health of the species [19].

For the overall SEUS habitat, the available information on right whales varies. There are descriptions of population status [20], distribution [8], [10], calving rates [21]–[23], relationship to environmental parameters [10], [24], [25], human impacts [19], survey methodology and efficacy [17], and impact-mitigation activities [16], [18], [26]. However, while behavior likely affects all of the foregoing, this topic has been little addressed.

There are few data on swim speeds of right whales. Swim speeds have been inferred from the linear distance between locations of nine satellite-tracked whales in 1989, 1990, and 1991 [27], [28]. The whales were initially tagged in the Bay of Fundy (New Brunswick, Canada) in the fall and displayed differences in speeds, movements, and areas occupied or visited. For the SEUS, swim speeds were similarly inferred from four satellite-tracked whales during the winters of 1996 and 1997 [29]. Lastly, also in the SEUS, using a VHF-radio tag on the adult, a mother-calf pair was tracked for 140 h in January 1999 [30]. Swim speed was inferred based on the position of the tracking vessel and the received signal strength from the radio transmitter (whales were often in visual range during daylight hours). For southern right whales off South Africa, swim speeds based on theodolite tracking from shore were obtained in October/November 1993 [31]. Subsequently, swim speeds and directional movements were described based on satellite tags deployed on 21 southern right whales off S. Africa in September 2001 [32].

In a section of the SEUS right whale critical habitat, a shore-based volunteer sighting network, working alongside experienced scientists, and supplemented by aerial surveys, has collected swim speed, movement, and behavioral data on North Atlantic right whales since 2001. In this southerly part of the Critical Habitat, where this study was based, commercial vessel traffic (and the corresponding jeopardy) is lower; although recreational vessel traffic, and the potential for harassment do exist. In this area, with some reduced potential for human impacts, and because of the nearshore occurrence, there is the opportunity for unobtrusive observation and monitoring. This paper describes observations for the southern portion of the SEUS Critical Habitat where direct observations were collected in an unobtrusive manner and where potential observer effects on whale behavior were absent. These behavioral characterizations provide new information on right whale biology, and provide input to predictive modeling and human-impact mitigation for this endangered species.

## Methods

### Study Area

**Figure 1:**
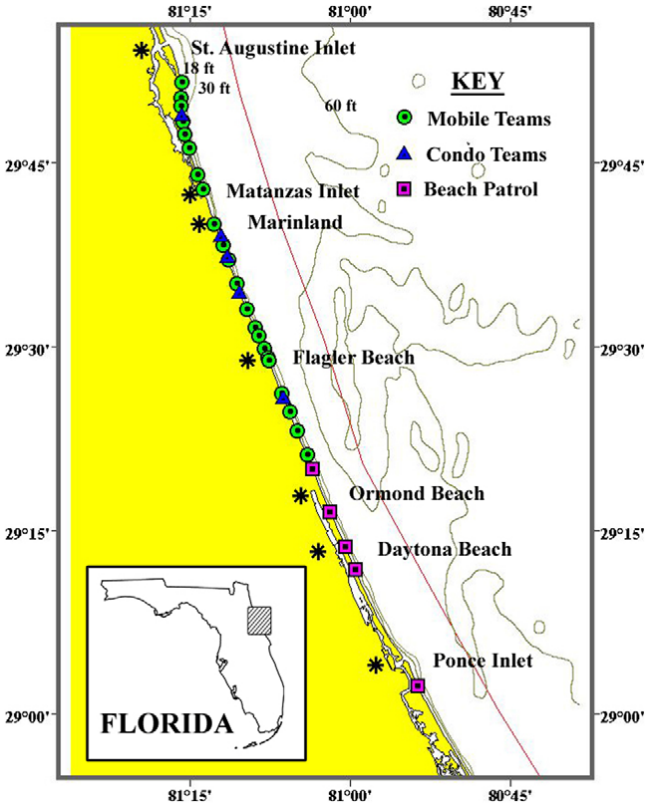
The study area showing the 33 lookout points used by the volunteer sighting network. The bathymetry (gray lines) was digitized from large-scale NOAA charts, and is equivalent to 5.5, 9.1, and 18.3 m. The solid red line is the boundary of the SEUS Right Whale Critical Habitat, which is 5 nm (9.3 km) from the coast in this area. A key feature of the study area is the narrowing of the nearshore zone of shallowest waters (<10 m) and increased depth closer to shore.

The study area was the nearshore waters of northeastern Florida (Figure 1). This area included about 120 km (65 nm) of beachfront/coastal waters, and was located from just north of the St. Augustine Inlet (∼30°00′ N) to just south of the Ponce de Leon Inlet (∼29°00′ N). The width of the area was approximately 3.7 km (2 nm) from the coast (*i.e*., the limit of visual detectability of right whales for shore-based observers). Maximum water depth was 18 m (60 ft) and for most observations was less than 14 m (45 ft), or about the length of an adult right whale.

### Observations

Sighting reports came from a network of approximately 200 trained volunteers. Sightings were reported to a central “hotline” maintained by the Marine Resources Council, Palm Bay, Florida. Each sighting report was relayed to local responders, and a response team that included experienced scientists was deployed. The response team carried portable GPS units (Garmin 12XL or similar), and digital cameras with long lenses (*e.g*., Canon EOS 20D with a Canon EF 600-mm image-stabilized f 4.0 lens fitted with a Canon 2X telextender). A monopod was used to support and stabilize the camera. Photographs were used for documentation of the sightings and for photo-identification of individual whales, with images matched to a catalog maintained by the New England Aquarium, Boston, Massachusetts [33].

Standardized protocols were followed for data collection. Bearings were measured using binoculars with built-in compasses (*e.g*., Nikon OceanPro 7X50 Model #7441). Angles at all locations were compared between two or more observers to avoid false readings caused by magnetic devices or metal objects. The resulting bearings were in agreement and accurate within one or two degrees. Ranges were estimated visually by experienced observers based on an initial series of calibration and training trials. A rangefinder that measured range to the sighting as a function of observer height-of-eye and declination angle below the horizon was used in the calibration trials. Height-of-eye from typical vantage points was 5 to 10 m. Visual estimation of distances from low-elevation shore stations can be difficult [34]. This improved with observer experience. In addition, recorded ranges and bearings were occasionally cross-checked by comparison with sighting positions recorded by research boats or aircraft that were near the whales. Distance or range agreements were typically ≤0.25 nm (0.5 km). In general, ranges were more susceptible to error, but had small effect on measurements of latitudinal or along-coast movement.

Data were gathered from approximately mid-December through mid-March. The volunteer sighting network included two components: 1) scheduled observers, and 2) opportunistic observers. The scheduled observers, typically teams of two to four volunteers, were of two types: a) mobile, and b) stationary. The mobile teams (total of four) met at 0800 h at a designated point, and traveled by vehicle to a series of lookout stations where a 15-min search was conducted at each. At the end of the series (typically five stations per team), they reversed the search and ended back at the original point. The stationary teams (total of five) maintained lookouts from dune walkovers or the balconies of shore-front buildings. In total, there were 33 lookout points along the coastline in the study area (Figure 1). In both cases, most watches were concluded by 1230 h (responses to reported sightings continued). The opportunistic observers were residents and/or workers who had been provided information and the hotline number; and reported whale sightings during the course of normal recreation or work. Opportunistic observers included the Volusia County Beach Patrol.

Swim speed and behavioral observations were collected during the course of a “follow” – defined here as a tracking period with one or more individual whales kept in sight from one to several hours. While sightings were occasionally reported from sea states ranging up to and including a Beaufort 5, the follows were conducted during sea states ≤ Beaufort 4. Follows were neither initiated nor continued when range or conditions precluded effective tracking. Comparison of photos taken at the beginning and end of a follow helped verify that the same individual(s) were tracked during interrupted observations (*e.g*., long dives). During follows, when whales moved along the coast, they were kept in sight by the coordinated efforts of two teams. From experience, we learned that the effective sighting range from typical shore vantage points was about 1200–1500 m (∼¾ nm). We also learned that to avoid moving whales being lost from view, a second team would be deployed in advance of the whales' movement. This was triggered as the whale reached a 30° horizontal or lateral angle from the perpendicular at the vantage point and approached the described distance range. Contact was maintained via cellular telephone. When the second team had the whales solidly in sight, the first team re-positioned. This “leap-frogging” was continued as necessary throughout the observation period – sometimes for an entire day. Particularly in the situation of elusive whales (lying low in choppy seas and/or increased submergence time), and/or a coastline with restricted vantage points, this close tracking was a necessary precaution. Photo-documentation and attention to distinctive markings, protocols that kept whales in sight, the low whale density, and the fact that mother-calf pairs generally isolated themselves, ensured that the follows were of the same whales. Positions for a given follow (incorporating times, ranges, and bearings) were plotted on large-scale NOAA navigational charts. The plotting of positions was done at the beginning and end of the observation period and intermediate positions whenever whales changed direction and/or behavior. Distances between positions were measured on the charts using standard nautical charting/plotting methods, and summed as necessary. From this, distance divided by time yielded speed.

Shore-based sightings (the principal data source for follows) were occasionally supplemented by those from survey aircraft (a Cessna 337 Skymaster operated by the Florida Fish and Wildlife Conservation Commission or an AirCam operated by our project). At times, sighting positions from observers on shore were relayed to the project's aircraft, which provided for additional individual photo-identification and behavioral characterization. Sighting positions obtained from the aircraft were also used to verify sighting distances estimated from shore, and on occasion, to add time and position data to follows.

### Mother-Calf Behaviors

Written notes and photographs taken during periods when mother-calf pairs were stationary were reviewed for common behaviors (observed on more than six occasions). The behaviors were recorded on handwritten data logs, and with aerial and shore-based video and still photography. Five of these behaviors are depicted graphically, and the sixth described textually.

### Definitions and Analyses

Throughout this report, sightings were categorized as mother-calf pair (MC), single or non-mother-calf pair (SPR), or group of ≥3 individuals (GRP). Most descriptions in this report are of mother-calf pairs. Mother-calf interactions and behaviors were defined as rest when both individuals were quiescent at the surface, and as play when the calf was actively swimming, lobtailing, flippering, and “romping” in the close vicinity of, against, and on top of the mother – and when the mother may or may not have been lobtailing or flippering. Nursing behavior was not visible from shore but was documented in aerial video and photographs, defined as when the calf was submerged perpendicular to the mother lying level at the surface, with the calf's head beneath the mother's abdomen in the area of the mammary slits. Because swim speed varied and sometimes included stationary periods of varying length, swim speed is reported as net swim speed based on distances between the endpoint locations for a given follow. While swim speed may be composed of both vertical and horizontal components, swim speed as observed during the follows reported here is defined as horizontal transit speed. The statistical analyses were conducted with SAS for Windows (version 9.1.3, SAS Institute, Inc., Cary, North Carolina).

## Results

During seven seasons, January 2001 through March 2007, 600 h of observations were made during the course of 109 follows by shore-based observers in the nearshore waters of northeastern Florida. These observations were occasionally supplemented by those from aircraft.

### Swim Speed

**Figure 2:**
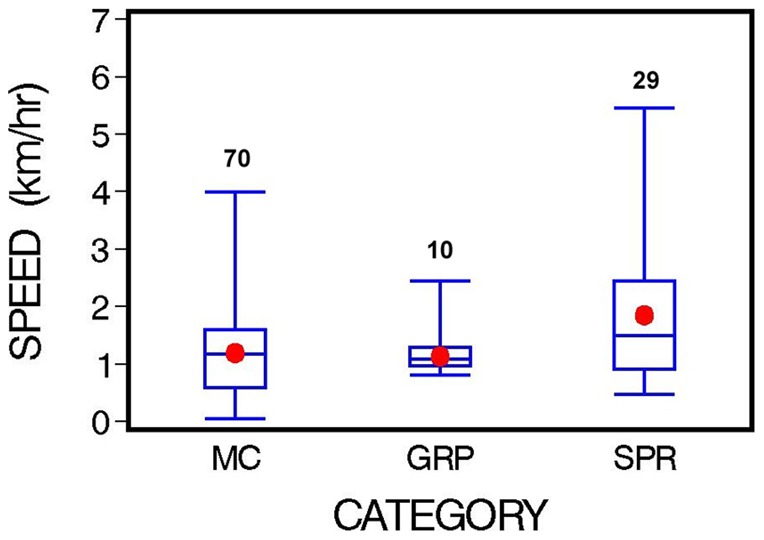
Swim speed for three categories of right whales (MC  =  mother/calf pairs, GRP  =  groups of ≥3, SPR  =  singles and non-mother-calf pairs) in nearshore waters of Florida, 2001–2007. Shown are the overall range, inter-quartile range (box), median (center line), mean (dot), and the number of observations (follows).

Swim-speed data for the three categories of sightings – MC, SPR, and GRP – suggest that the SPR category had the fastest mean swim speed and greatest variability (Figure 2, Table 1). The other two categories had similar mean speeds, but the small sample of GRPs had less variability. An exploratory ANOVA (Kruskal-Wallis Test) for the three categories closely approached statistical significance (*P* = 0.054). The follow-up pair-wise comparisons (Duncan's Multiple Range Test, α = 0.05) showed that MCs and GRPs were not significantly different from each other, while the SPR class was significantly faster (Table 1). Additional summarized values for the examination of swim speed are provided in Table 2. In aggregate, MC swim speeds of greater than 1.9 km/h (1.0 kn) were observed on only 11 occasions (15% of the follows in the MC category). Slower speeds were common across all categories. Of the total 109 follows across all three categories, swim speeds ≤0.9 km/h (0.5 kn) occurred in 36% of all records, and ≤1.9 km/h (1 kn) in 79% of all records.

**Table 1:** Swim speeds (km/h) for three categories of right whales in coastal waters of northeastern Florida, 2001–2007. (See Fig. 2 for category definitions.)

Category	N*	Hours	Mean	Range	SD	SE	Median	Wt. Mean**
MC	70	398.2	1.20	0.05–4.07	0.76	0.09	1.17	1.15
SPR	29	141.8	1.86	0.48–5.37	1.27	0.24	1.50	1.84
GRP	10	57.4	1.26	0.81–2.44	0.50	0.16	1.09	1.18
Sums	109	600.4						

Time over which speeds were calculated were MC, range = 0.8–10.6, mean = 5.7, median = 5.5; SPR, range 1.2–9.1, mean = 4.9, median = 4.7; and GRP, range = 1.3–8.7, mean = 5.8, median = 6.3 hr.

* Number of follows.

** Weighted mean, weighted by follow duration.

**Table 2:** Summary of swim-speed values for right whales in coastal waters of the SEUS.

Item	Swim-speed (km/h (kn))	Notes
Median swim speed across all categories (weighted by the number of observations in each category	1.3 (0.7)	
Median swim speed for all MC observations	1.1 (0.6)	398 h of observations
Fastest swim speed for a MC pair	4.1 (2.2)	Southbound female and calf on 4 December 2005
Fastest swim speed by a non-MC pair	3.6 (1.9)	Catalog #2660 and an unidentified individual on 21 March 2004
Fastest swim speed for all categories	5.4 (2.9)	Southbound single sub-adult on 13 February 2007

**Figure 3:**
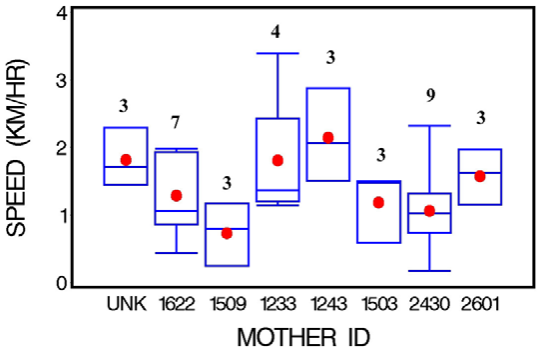
Swim speed for eight mother-calf pairs of right whales with at least three follows in nearshore waters of Florida, 2001–2007 (format as in Fig. 2).

Several MC pairs were each followed on multiple occasions. Speed varied both between and among identified individuals. Were the swim speeds of individual MC pairs similar or different? Of the total (27) of identified MC pairs followed, 17 (63%) were followed only on a single occasion, and three (11%) on two occasions. The remaining seven were followed on three to nine occasions each, and one other pair where the photographs were inadequate for individual identification was followed three times. For these remaining eight pairs with ≥3 follows, the swim speed data are summarized and compared in Figure 3 and Table 3. There was no statistically significant among-pair variability (Kruskal-Wallis *P* = 0.135).

**Table 3:** Swim speeds (km/h) for eight different mother-calf pairs of right whales in coastal waters of northeastern Florida, 2001–2007.

ID #	N	Tot hrs	Range	Mean	SD	SE	Median	Wt mean
Unk*	3	21.5	1.48–2.22	1.79	0.39	0.22	1.67	1.74
1233	4	29.8	1.11–3.33	1.81	1.03	0.52	1.39	1.55
1243	3	17.8	1.48–2.96	2.16	0.75	0.43	2.04	2.14
1503	3	22.5	0.56–1.48	1.17	0.53	0.31	1.48	1.20
1509	3	22.1	0.20–1.11	0.68	0.47	0.27	0.74	0.62
1622	7	29.4	0.37–2.04	1.28	0.64	0.24	1.11	1.22
2430	9	52.7	0.17–2.32	1.07	0.63	0.21	1.02	1.00
2601	3	19.7	1.15–1.96	1.57	0.41	0.24	1.61	1.46

(Parameters as in Table 1.).

* photographs were inadequate to identify this female.

Three of these cases provided some description of the occurrence of stationary periods versus active swimming. Female #1622, observed in both the 2002 and 2005 seasons, with 29.4 h of total observation, had a median swim speed of 1.1 km/h (0.6 kn), and a range of 0.4–2.0 km/h (0.2–1.1 kn). On 21 February 2002, #1622 and calf were stationary for 5.5 h. During the remaining six follows of #1622 and calf on other days, net swim speed was between 0.9 and 2.0 km/h (0.5 and 1.1 kn). Female #1509, with calves, was observed in both the 2001 and 2004 seasons. On 6 February 2001, the pair was stationary (moving only 1.3 km or 0.7 nm) for the entire 9 h observation period. In 2004, for 13 h of observation during two follows on two days in February, swim speed was 0.9 km/h (0.5 kn). Female #2430 and calf, observed in the 2007 season with 52.7 h of total observation, had a median of 1.0 km/h (0.6 kn), and a range of 0.2–2.3 km/h (0.1–1.3 kn). On 3 January 2007, #2430 was stationary for 4.3 h, and during the eight other follows, the net swim speed was between 0.5 and 2.3 km/h (0.3 and 1.3 kn). Another long stationary period by a MC pair was 7.5 h by female #1245 and calf on 13 February 2005. For a given day, the occurrence of stationary periods versus forward swimming is a variable. For the two examples of MC pairs with numerous follows (#s 1622 and 2430), we explored whether swim speed varied by date, but no correlation could be detected.

### Mother-Calf Behavior

**Figure 4:**
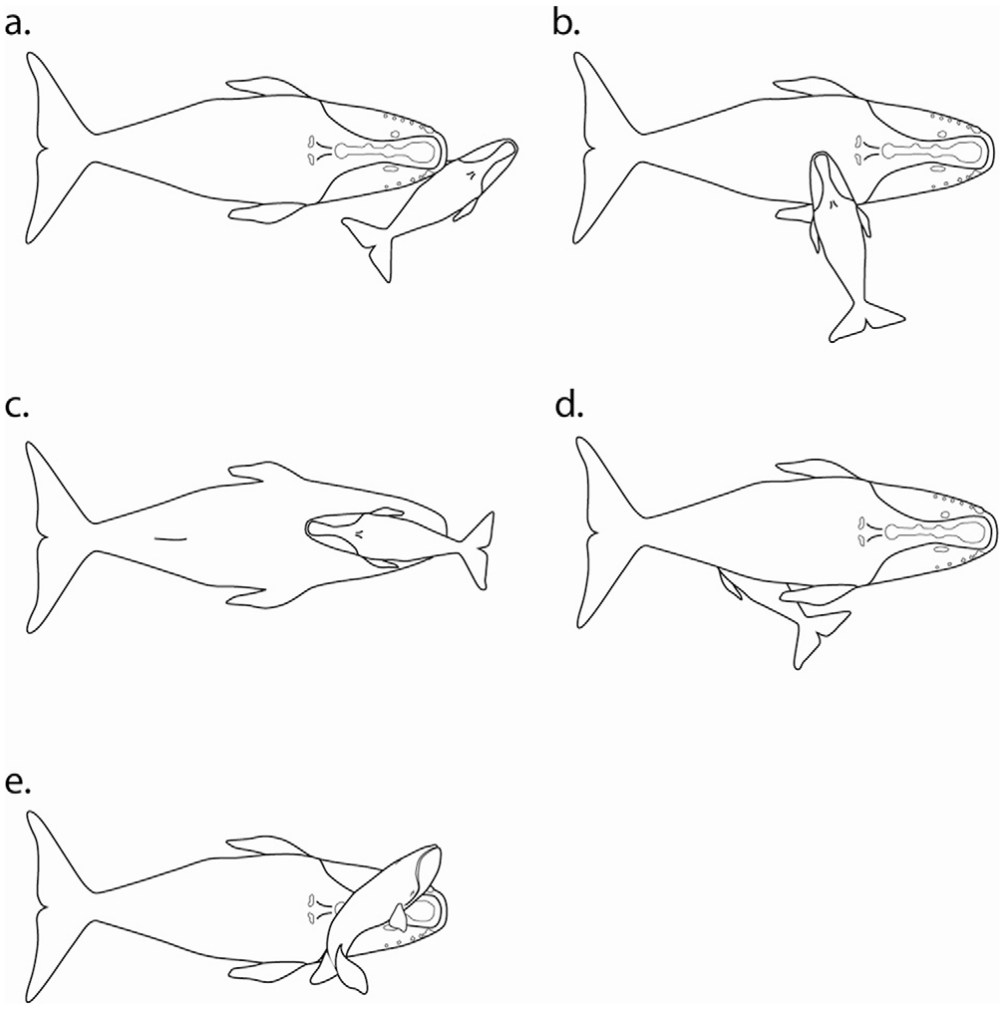
Mother-calf interactions and behaviors: a. Calf positioned diagonally with mother's chin touching calf. b. Calf's chin resting on mother's back. c. Mother inverted (belly up), calf swimming in the opposite direction. d. Calf apparently nursing. e. Calf “romping” across mother's head. (Graphics by P. Oberlander, based on photos).

As described, swimming was rarely continuous in MCs, but rather, the measured net speeds included stationary periods. During periods when mother-calf pairs were stationary, two general classes of behaviors were observed: typical diving behavior, and surface-based mother-calf interactions. Periods of diving and submergence provided little information. On the other hand, periods of mother-calf interactions at the surface were common, lasting from less than an hour to several hours. These interactions between mother and calf often included behaviors that were recorded across individuals, days, seasons, and years (Figure 4). These surface behaviors ranged from quiet contact (a, b, and c), to apparent nursing (d), and boisterous play (e). Calves were more active than mothers.

### Direction of Movement

**Figure 5:**
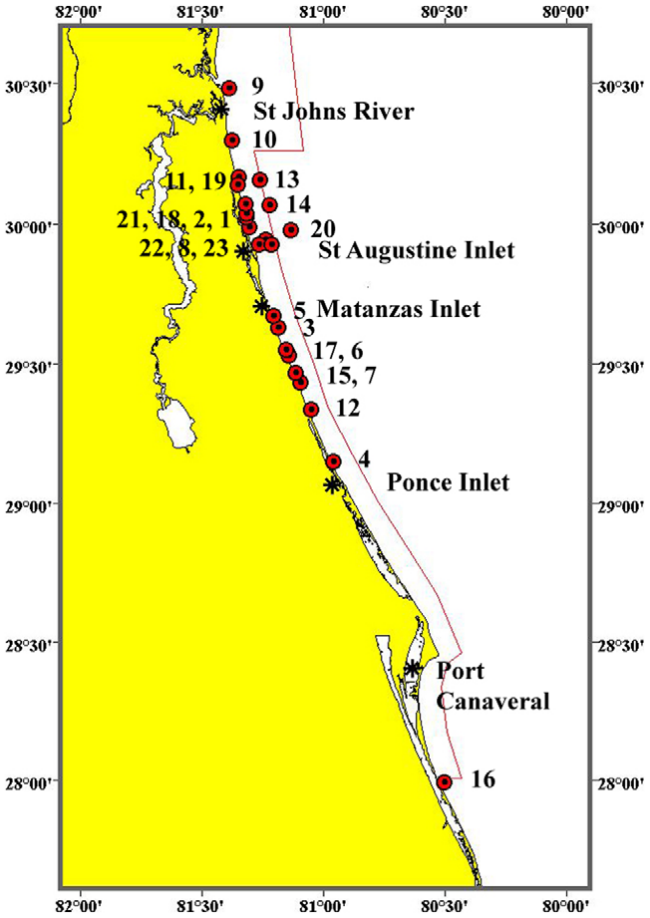
Sightings for female #2430 and calf during a nine-week period, 27 December 2006 to 24 February 2007, including several contributed by the Florida Fish and Wildlife Conservation Commission. During this period the pair swam south four times and north four times, traversing 158 nm (292 km), and passing by inlets and channel entrances on several occasions (points of possible increased risk to whales). Sighting frequency is related to effort (sighting effort was less south of Ponce Inlet). The movement can be tracked by following the sequence of numbers. Key: 1-12/27/06, 2-12/28, 3-12/29, 4-12/30, 5-1/03/07, 6-1/04, 7-1/05, 8-1/07, 9-1/09, 10-1/10, 11-1/16, 12-1/21, 13-1/27, 14-1/30, 15-2/02, 16-2/05, 17-2/12, 18-2/13, 19-2/18, 20-2/20, 21-2/21, 22-2/22, 23-2/24.

The observation of along-coast movements revealed another behavior – the reversal of direction. Of the 70 MC follows, most were sighted on a single occasion (*i.e*., one focal follow per identified mother and calf). Movement was generally, but not always, parallel to the coast. For the 70 follows, 37 MCs (53%) swam south, 20 (29%) swam north, and 13 (19%) were stationary or changed direction one or more times during the course of the follow. The reversal of direction during a follow was observed on several occasions for MCs. Four examples follow:

1. On 31 January 2001, a MC pair (the one not identified/matched to the catalog) sighted initially at 1030 h at 29°15.4′ (off Daytona Beach), swam north until 1820 h and 29°22.1′. They were re-sighted the next morning at 29°31.0′ (18.5 km or 10 nm to the northwest) and continued to swim north until 1430 h. Once they reached 29°34.3′, the pair made an abrupt U-turn, and swam south until 1745 h and 29°31.9′ (just 1.85 km or 1 nm from the first position of the day). The same whales were re-sighted the following morning at 0945 h and 29°23.0′ (9.7 nm from the last position of the previous day) and continued to swim south until 1514 h. The last sighting was at 29°16.8′ (off Ormond Beach), when deteriorating weather precluded further observations. During the three days, the pair swam 38.3 km (20.7 nm) north, then 35.2 km (19.0 nm) south, and was last sighted only 3.3 km (1.8 nm) from the position of the initial sighting.
2. At 1025 h on 5 February 2004, mother #1509 and her calf were sighted at 29°33.7′, swimming south. At 1155 h, the pair changed direction and swam west-northwest, angling in toward shore. At 1215 h, the pair was within 0.5 km (¼ nm) of shore at 29°33.0′. Shortly thereafter, they swam north, maintaining direction until the last sighting of the day at 1643 h and 29°35.4′. The final sighting on this day was 4.3 km (2.3 nm) from the first. In 6.3 h, the pair had several stationary periods and swam 7.4 km (4 nm) in a triangular course with a net speed of 1.1 km/h (0.6 kn).
3. Another perspective is provided by using a fixed reference point over the course of several follows. In one example, mother #2430 and calf were sighted on 19 occasions between 27 December 2006 and 24 February 2007 in the area between 29°59.2′ and 27°59.4′ (240.5 km or 130 nm in latitudinal extent) (Figure 5). The pair swam back-and-forth past 29°28.8′ (Flagler Beach) on six occasions.
4. In a final example, female #1622 and her calf were sighted five times between 17 February and 6 March 2002, and traveled south and north in a 74-km (40 nm) section, passing by the St. Augustine Inlet and Matanzas Inlet on four different occasions.

## Discussion

### Observational Method

A unique situation – right whales close to shore and monitored during multiple years by a shore-based sighting network – provided observations that contribute to conservation biology and recovery efforts. Shore-based observations of right whale behavior have been reported from other locations, including South Africa [31], Australia [35], and Argentina [36]–[39]. Here, for the first time, observations from the southeastern United States are described. As in several other studies, shore-based observations were supplemented by those from an aircraft and drew on the invaluable resource of a catalog and associated database [33]. Some constraints were imposed by temporal and spatial variability in sighting effort, as well as some limitations on data resolution, and resulted in some descriptions that are qualitative. While height-of-eye in Argentine studies was 46 m above sea level and that of the South African study 80 m, our height-of-eye was typically 5 to 10 m – limiting the observer's range of view. However, the temporal and spatial coverage provided by the volunteer network (“large area, few whales, many eyes”), the use of cameras with long lenses and the aircraft, as well as the protocols that aided extended follows, provided for unique and useful data.

### Swim Speed

As described, previous swim-speed reports have mostly been from tagged animals. For the SEUS, four individuals were satellite-tracked during the winters of 1996 and 1997 [29], and female #1612 and her calf were tracked using a VHF radio tag in January 1999 [30]. In that study [30], a mother-calf pair meandering within the habitat appeared to swim at between 0.4 and 1.1 km/h (0.02–0.6 kn), within the range reported here.

Studies in other habitats and locations describe comparable findings and also distinguish between non-migrating (“seasonal residence”) and migrating speeds. For the nine right whales initially tagged in the Bay of Fundy 1989–1991, the average speed was 2.7 km/h (1.5 kn) but differed widely among tagged whales [27]–[28]. For the subset of whales that remained within the Bay, mean net swim speeds were 1.1 km/h (0.6 kn). For coastal waters off South Africa in 1993, (the only other known direct swim-speed observations), overall mean speed was 1.7±0.9 km/h (0.9±0.5 kn) with a range of 0.4–3.6 km/h (0.2–1.9 kn) [31]. For four MCs, mean speed was 2.0±1.2 km/h (1.1±0.6 kn) with a range of 0.4 to 3.3 km/h (0.2 to 1.8 kn). For these same waters, and based on satellite-tracking, mean net speeds for combined mother-calf pairs and several single individuals ranged from 1.0 to 2.8 km/hr (0.5 to 1.5 kn) (SD  = 5.9 km/hr, n = 11) [32]. These reports (for different areas, different methods, and different years) are consistent with present findings – where the median within-habitat swim speed for all categories was 1.3 km/h (0.7 kn) and generally ≤1.9 km/h (1 kn).

On the other hand, whales in the between-habitat transitory or migratory mode have faster net swim speeds. Right whales that left the Bay of Fundy had higher average speeds (3.5 km/h or 1.9 kn) than those that remained within the Bay [28]. Likewise, whales that departed the South African coast for offshore areas displayed mean net speeds of 3.3 km/h or 1.8 kn [32]. For the SEUS, MC #2503 was recorded moving southbound rapidly on 4 December 2005, enroute to the Gulf of Mexico [40] and a pair that included female #2660 was documented moving northbound on 21 March 2004, and sighted off Georgia three days later (pers. comm., M. Zani, New England Aquarium). These net speeds were approximately twice –3.7 to 5.6 km/hr (2 to 3 kn) – than what was described previously as the within-habitat speeds. For right whales, in the SEUS and elsewhere, the suggestion is that within-habitat swim speeds are different from between-habitat or transiting swim speeds. While these two examples are from the beginning and end of the calving season, the whales described in this study had different arrivals and departures, as well as different calving dates, so distinctions between resident and migrating speeds will be difficult to generalize, as behaviors and movements may be temporally variable for different individuals. A number of factors are likely involved, and further study is warranted.

### Stationary Periods and Mother-Calf Interactions

When swimming ceased, a period of diving and submergence sometimes occurred. Because whales were not generally visible during these periods, this study did not address this topic. One behavioral aspect, however, is relevant to surveys and detection. At times, the mothers were submerged and the calf remained alone at the surface. In a previous study [17], when surface and dive times were described, calves had shorter dives than their mothers and spent a greater portion of time at the surface Calves were alone at the surface 22% of the time. (This item was not quantifiable in the present study.) When this occurs, the considerably smaller calf may reduce detection by aerial surveys as well as shore spotters.

The more readily observed behavioral state was surface-based mother-calf interactions during stationary periods. One report described that “female right whales with calves can spend prolonged (up to an hour) periods at the surface either moving slowly or not at all” [30]. With additional data, it can be reported that these prolonged stationary periods are sometimes as long as 9 h. During stationary periods at the surface, the mother was often seen with her chin against the calf's body where the calf was positioned diagonally in front of the mother. At other times, the calf positioned its chin on the back or belly of the mother. This chin contact may be significant. Cetaceans have a well-developed tactile sense [41] and the chins of balaenids have concentrations of hairs and sensory papillae [42] – enhancing the contact. More broadly, Our observations in the SEUS were consistent with those for southern right whales, where mothers and calves were almost continuously in physical contact with each other [38]. As described for swim speeds, we note the similarities in several of the MC behaviors to those for other right whale species in other hemispheres and different decades [39].

These MC behaviors and tactile contact likely contribute to a mother-calf connection – a connection that likely includes a communicative function. Behavioral events that may seem trivial in an individual instance may carry important information, including communicating the internal state of the individual [43]. The mother-calf interaction almost certainly includes teaching and learning [44]–[46]. It is possible that behavior that contributes to life success is imparted and acquired during these first months of a calf's life in the SEUS habitat. At times both mother and calf were quiescent. At other times, the calf was boisterous and the activity could be described as play. This more vigorous activity is essential for later survival [39], as it may function to develop motor skills useful in social, reproductive, and feeding contexts. During the calf's first months of life, it is exercised in breathing and swimming and muscles are developed. This may be related to predator avoidance [47] and the northward migration. Collectively, these behaviors are a highly important part of their biology [43]. For management considerations, these descriptions of mother-calf behaviors reinforce the importance, complexity, and dimensions of MC interactions, and likewise reinforce the prudent awareness and caution required in situations when harassment from humans may occur. A further aspect of the mother-calf behavior with relevance to management is the importance to the mother of conserving resources during her period of fasting while nursing her calf [39]. This need to conserve energy should likewise be considered when the potential for harassment may occur.

### Movements and Direction of Travel

Within a habitat, back-and-forth traveling movements appear common [27]–[30], [32]. Several U-turns or back-tracks were observed in this study. For the southern part of the SEUS habitat, these movements appear linear and parallel to shore, generally north-south in direction. (As described previously, from the shore-based vantage points, descriptions of inshore/offshore movement perpendicular to the shore are more limited.) For management considerations, these back-and-forth movements, when they do occur, may be related to “exposure.” That is, if there is a point in the habitat that may present jeopardy or risk, an individual whale may be exposed on more than one occasion. In the two examples described above, MC #1622 and MC #2430 repeatedly passed by a number of inlets. The effect of this behavior is that right whales may be exposed on multiple occasions to the commercial and recreational vessel traffic associated with these channels and inlets. When exposure is increased, jeopardy from whale-vessel interaction or collision is likewise increased [48], [49].

### Implications for Management and Modeling

Information that contributes to conservation biology accumulates gradually. For an endangered species such as the North Atlantic right whale, all advances are significant. This is particularly true for behavior, where observations and data collection can be challenging. This paper contributes information to right whale natural history in a portion of the southeastern U.S. right whale critical habitat. The understanding of right whale swim speed, behavior, and movement has application to survey and search effort, to mitigation of human impacts, risk models, and ultimately, to right whale conservation and continuing recovery.

Among the dimensions to this study is the involvement of more than 200 citizens in a shore-based sighting and monitoring network – contributing effort and resources where otherwise there may be few or none. Because the volunteers interact with neighbors, friends, and others, an expanding knowledgeable and engaged citizenry is generated. Additionally, unlike inferences drawn from indirect and unattended data collection (*e.g*., satellite tagging), the observations here are direct and uninterrupted for extended periods. At the same time, visual follows also have limitations, and do not account for night-time, sub-surface, and poor sighting conditions. However, these observations add detail that would otherwise go unknown (*e.g*., stationary periods and mother-calf behaviors) Further, the consistency between the direct measurements reported here and the indirect measurements resulting from satellite tracking adds confidence to the satellite data. Lastly, unlike circumstances where research platforms are in close proximity to the whales for periods of time and/or there may be physical contact related to sampling or tagging, the observations here were unobtrusive, so unbiased behavior is reported.

Throughout, we have been cautious in interpreting our findings and hesitant in extending conclusions beyond the current data. In several cases, the description of behaviors is qualitative rather than quantitative. Several questions can be identified for future research: Do behaviors change with calf age? Does swim speed change as a calf ages? Do tides and currents influence the swim speed and/or travel direction? Can shore-based observations aid in assessing the impact of research platforms, boats and aircraft, on behavior? To what extent do right whales occur farther offshore (beyond 5 nm) south of the narrowing of the critical habitat at 30°15′ N?

This study has provided quantitative descriptions of swim speeds. These values are consistent with those reported for other methods and other geographic areas, and may be of use to managers and modelers. For example, if swim speed is known, then distance traveled during a given time period can be estimated. In addition (taking into account the possible meandering or back-tracking behavior), areas can be established for re-locating or re-sighting efforts, for a caution alert or safety radius, or for a potential risk warning. Other behavioral descriptions are more qualitative and, while providing useful insights, do not provide specific values. In all cases, variability is evident throughout. While we often search for predictions, patterns, and means, the message here is also about variability and the behavioral characteristics of individual whales. This will necessarily result in broader statements and appropriate uncertainty for both managers and modelers.
